# The Effects of Lumbar Delayed Onset Muscle Soreness on Clinical, Biomechanical and Neuromuscular Outcomes: A Systematic Review and Meta‐Analysis

**DOI:** 10.1002/ejp.70264

**Published:** 2026-04-11

**Authors:** Julien Ducas, Clémentine Véret, Émilie Gauthier‐Wong, Martin Descarreaux, Jacques Abboud

**Affiliations:** ^1^ Department of Anatomy Université du Québec à Trois‐Rivières Québec Canada; ^2^ Groupe de Recherche sur les Affections Neuromusculosquelettiques (GRAN) Université du Québec à Trois‐Rivières Québec Canada; ^3^ Department of Human Kinetics Université du Québec à Trois‐Rivières Québec Canada

**Keywords:** DOMS, experimental pain, low back pain, pain, soreness, trunk

## Abstract

**Background and Objective:**

Low back pain is multifactorial, making it difficult to isolate pain‐related motor adaptations. Experimental pain models may help clarify these mechanisms. This systematic review evaluates lumbar delayed onset muscle soreness (DOMS) as a movement‐evoked pain model and its effects on clinical, biomechanical and neuromuscular outcomes.

**Databases and Data Treatment:**

The meta‐analysis (PROSPERO: CRD420251051399) included studies of healthy adults with experimentally induced DOMS, with outcomes assessed within 5 days under the influence of DOMS. Databases (MEDLINE, SCOPUS, CINAH) were searched (9/12/2025), with manual screening. Independent study selection, data extraction and risk‐of‐bias assessment (NIH Quality Assessment Tool) were performed. Random‐effects meta‐analysis calculated standardized mean differences (SMDs). Sensitivity analyses and GRADE (Cochrane) assessed robustness and evidence certainty.

**Results:**

Eighteen studies were included (452 participants). DOMS significantly increased low back pain intensity (SMD: Day 1 with DOMS = 1.12, Day 2 = 0.94, Day 3 = 0.69, Day 4 = 0.49) and soreness (Day 1 = 1.94, Day 2 = 1.66, Day 3 = 0.88, Day 4 = 0.59). Pressure pain thresholds decreased significantly (Day 1 = −0.47, Day 2 = −0.44). Trunk extension maximal voluntary contraction decreased significantly (Day 1 = −0.67, Day 2 = −0.95, Day 3 = −0.84, Day 4 = −0.38), while trunk flexion range of motion and flexion relaxation ratios remained unchanged.

**Conclusions:**

Lumbar DOMS replicates low back pain features, movement‐evoked pain, increased sensitivity and reduced muscle function, providing a non‐invasive model to study short‐term neuromuscular adaptations in a controlled setting.

**Significance Statement:**

This review highlights lumbar DOMS as a safe, non‐invasive model to study short‐term neuromuscular adaptations to pain to better understand pain mechanism. This synthesis provides foundational work for future studies using DOMS to explore LBP mechanisms. By clarifying its strengths (e.g., ecological validity for acute pain) and limitations (e.g., short duration, lack of pain‐related psychological factors), we aimed to guide more standardized applications of this model in pain research.

## 1 Introduction

Low back pain (LBP) is the leading cause of disability worldwide, affecting over 600 million people in 2020, with prevalence projected to rise (Ferreira et al. 2023). The Lancet research priority series emphasized that LBP is a complex, multifactorial condition influenced by psychological, social, biophysical and pain‐processing factors (Buchbinder et al. 2018; Clark and Horton 2018; Foster et al. 2018; Hartvigsen et al. 2018).

Characterizing LBP motor adaptations is challenging, and comparisons between individuals with LBP and asymptomatic individuals are influenced by confounding factors such as heterogeneous pain sources, variable pain duration, psychological factors and individual compensatory strategies, all of which complicate interpretation of findings (Otero‐Ketterer et al. 2022). Several experimental pain models have been developed over the years to address these challenges and better understand pain motor adaptations. These models allow controlled pre‐post comparisons within the same individual, thereby isolating the specific effect of pain on motor adaptations and reducing inter‐individual variability. While experimentally induced pain does not fully replicate the characteristics of musculoskeletal disorders, developing models that closely mimic clinical LBP is essential. In most experimental studies, LBP is induced using pain models that produce short‐lasting and constant pain (e.g., hypertonic saline solution or heat pain) (Arendt‐Nielsen et al. 1996; Dickx et al. 2010; Dubois et al. 2011; Hodges et al. 2013; Tsao et al. 2010). A limitation of these models is that pain intensity cannot be modulated based on the motor behaviour used to perform a task, as it naturally occurs in chronic LBP conditions.

Delayed onset muscle soreness (DOMS) is often considered a more representative model of musculoskeletal pain, as it induces movement‐evoked pain resulting from muscle damage and inflammation (Cheung et al. 2003; Farias‐Junior et al. 2019). DOMS typically follows intense physical activity, especially following eccentric contractions (Clarkson and Hubal 2002; Lewis et al. 2012; Newham et al. 1983). It usually peaks around 24–72 h following physical activity, with increased pain and soreness in the damaged muscle (Abboud et al. 2019; Cheung et al. 2003; Cleak and Eston 1992). Unlike other experimental pain models, DOMS does not require invasive procedures or external stimuli, reducing the risk of confounding factors in the interpretation of neuromuscular adaptations to pain.

Despite growing interest in DOMS as an experimental pain model for clinical LBP, the literature remains scattered, with most studies typically focusing on single outcomes such as strength loss, changes in muscle activation patterns, movement alterations, or clinical features. A comprehensive understanding of how lumbar DOMS influences clinical, biomechanical and neuromuscular outcomes is needed to validate DOMS as a useful experimental model to mimic clinical LBP.

### 1.1 Research Question

In healthy individuals, what are the effects of experimentally induced lumbar DOMS, compared to a pain‐free condition, on clinical, biomechanical and neuromuscular outcomes related to trunk function?

### 1.2 Objective

This review aimed to evaluate the effects of lumbar DOMS on clinical, biomechanical and neuromuscular outcomes related to trunk function.

## 2 Methods

This review was conducted following a predefined protocol registered in the International Prospective Register of Systematic Reviews (PROSPERO; CRD420251051399) on 30/05/2025. The protocol was developed in accordance with the Cochrane Handbook (Chandler et al. 2019) and the PRISMA‐P preferred reporting guidelines (Moher et al. 2015; Page et al. 2021).

### 2.1 Eligibility Criteria

The PICOS (P: Population; I: Intervention; C: Comparison; O: Outcome(s); S: Study design) framework has been used to inform the eligibility criteria for inclusion and exclusion of studies (Shamseer et al. 2015). Detailed specifications for each PICOS component are provided in the following sections.

### 2.2 Population

Studies must involve healthy, back pain‐free adults aged between 18 and 65 years.

### 2.3 Intervention/Exposure

Eligible studies must experimentally induce DOMS in the lumbar region and assess its effects on clinical, biomechanical and neuromuscular outcomes related to trunk function. DOMS had to be induced without being combined with other interventions, to avoid confounding effects.

### 2.4 Comparison

To address the review objective, studies must include within‐subject comparisons of clinical, biomechanical and neuromuscular factors related to trunk function between conditions with and without lumbar DOMS.

### 2.5 Outcomes

Studies must evaluate at least one of the following outcome categories in the lumbar region: clinical outcomes (e.g., LBP intensity, perceived soreness, lumbar stiffness, pressure pain threshold (PPT)), biomechanical outcomes (e.g., trunk extensor maximal voluntary contraction (MVC), torque steadiness, muscle endurance), or neuromuscular outcomes (e.g., flexion‐relaxation phenomenon, muscle activity amplitude, spatial distribution of muscle activity). To align with the peak manifestation of lumbar DOMS symptoms and given that the duration and resolution of DOMS can vary between individuals and protocols, lasting up to 5 days post‐exercise, data collected up to day 5 were extracted and analyzed (Abboud et al. 2019; Cheung et al. 2003; Cleak and Eston 1992). Extending the analysis to this time point allowed for the examination of the temporal evolution and recovery of DOMS‐related outcomes while still remaining within the generally accepted timeframe during which lumbar DOMS symptoms are relevant (Abboud et al. 2019). Evaluations of systemic changes (e.g., inflammatory blood markers, alterations in brain structures, or central pain mechanisms) were not extracted because the focus of this review was on adaptations within the lumbar region rather than on general physiological or central responses to DOMS.

### 2.6 Studies

Experimental studies with a within‐subject or crossover design were considered for inclusion. Observational studies, case reports and studies involving clinical LBP were excluded. Studies were considered if they were published in English or French.

### 2.7 Information Sources

A comprehensive search for information sources was conducted covering the period from the inception of the topic until 9/12/2025. The search process was facilitated by a university librarian, who reviewed the selected databases and refined the search strategy, including the selection of keywords in the search. The databases selected for this search include MEDLINE, SCOPUS and CINAHL. Alongside the database searches, a manual review of journals relevant to the domain was conducted (e.g., Pain, Journal of Pain, European Journal of Pain, Journal of Electromyography and Kinesiology, Journal of Neurophysiology). Grey literature (e.g., reports and conference abstracts) was searched by J.D. in the OpenGrey database. Additionally, the reference lists of all included studies were manually screened by J.D. to identify further relevant sources. Theses were not included, as they often lack rigorous peer review and may not meet the methodological standards required for this review.

### 2.8 Search Strategy

The search strategy was designed around three primary domains to address the study objective: DOMS, adaptations (clinical, biomechanical or neuromuscular outcomes) and lumbar region. Terms commonly associated with each domain were combined using the Boolean operator “OR”. The three main domains were then interconnected using the Boolean operator “AND”. Tailored search strategies using Medical Subject Headings (MeSH) were applied where applicable. To ensure consistency, the search strategy was tailored to each database while maintaining uniformity across platforms. The search strategy implemented via the EBSCO interface is presented in Supporting Information S1.

### 2.9 Data Management

Management of articles, citations, abstracts and full texts of relevant studies was conducted on Covidence software (Veritas Health Innovation, Melbourne, Australia. Available at www.covidence.org), where any duplicate studies were automatically removed before the screening process began. Covidence was also used for the organization and tracking of each study through the screening, eligibility and data extraction phases.

### 2.10 Selection Process

Database searches were conducted by one team member (C.V.). Titles and abstracts were reviewed independently by three team members (J.D., C.V. and E.G.‐W.), with studies categorized in the Covidence software under three options: no, maybe and yes. In the event of conflict between two reviewers on the inclusion of studies, the third member assessed the disputed articles. All articles marked as “maybe” or “yes” were reviewed in full by J.D., C.V. and E.G.‐W. to finalize the total number of articles eligible.

### 2.11 Data Extraction Process

Data extraction was conducted by J.D., C.V. and E.G.‐W. independently. In the event of a conflict between two reviewers, any disagreements were resolved by the third member. A data collection form was developed in Microsoft Excel by all the team members and pilot tested. For data only available in figures, Plot Digitizer software (PlotDigitizer, https://plotdigitizer.com, Version: 2.6.11b) was used, as it has been shown to offer higher inter‐rater reliability than manual extraction (Kadic et al. 2016). Authors of studies were contacted for unpublished results that were considered important for data extraction.

### 2.12 Data Item

Extracted items in included studies followed the PICOS framework and are presented in Table 1.

**TABLE 1:** Extracted item in included studies.

Articles information	Authors
	Title
	Publication year
	Country
	Study design
	Study objectives
Population	Number of participants
	Gender or sex
	Age
	Weight
	Height
	Body mass index
Intervention	DOMS protocol
	Assessment timepoints
	Task details
Comparator	Condition without DOMS (baseline/control condition) for within‐subject comparison
Outcomes	Outcome domains (clinical, biomechanical or neuromuscular)
	Outcome measures (e.g., muscle activity amplitude, force steadiness, range of motion and pain intensity)
	Measurement tools (e.g., EMG, force plates, 3D kinematic cameras and numerical rating scales)

### 2.13 Quality Assessment

Quality of individual studies was evaluated based on the following criteria: clearly stated study objectives, eligibility criteria, representativeness of the study population, enrolment of all eligible participants, sufficient sample size, clear description of the intervention, specification of outcome measures, blinding of outcome assessors, completeness of follow‐up and appropriate statistical analysis of outcomes pre‐ and post‐intervention (National Heart and Institute 2014). This assessment was conducted independently by C.V. and E.G.‐W., with J.D. consulted in case of disagreement, using the Quality Assessment Tool for Before‐After (Pre‐Post) Studies With No Control Group developed by the National Heart, Lung, and Blood Institute (NIH) (National Heart and Institute 2014). This tool was used to evaluate the risk of bias and internal validity and was selected based on the recommendations of Ma et al. (2020), who identified it as the most appropriate tool for evaluating before‐after studies without a control group. All of the included studies used a pre‐post design without a control group, except for two studies that also used a crossover design (Houle et al. 2020; Lee et al. 2020). For consistency, we used the same NIH quality assessment tool across all studies, as no specific NIH tool exists for crossover designs. Moreover, the crossover studies involved the same participants across conditions, making the pre‐post assessment criteria applicable. The authors rated the quality of each study as “poor”, “fair”, or “good” based on the perceived risk of bias (J.D. and E.G.‐W.). Disagreements were resolved through consultation with a third author (J.A.) (National Heart and Institute 2014). The identified risk of bias did not exclude a study from being included in the data synthesis.

### 2.14 Data Synthesis

Results were synthesized quantitatively whenever studies were sufficiently homogeneous in outcomes, experimental conditions and timing (i.e., comparable measures taken before and under the influence of DOMS). If studies addressing an outcome were too heterogeneous to justify pooling, a narrative synthesis was provided. When pooling was appropriate, effect sizes were computed from within‐subject (pre‐post) change scores. Effect sizes were calculated as standardized mean differences (SMDs) using within‐subject change scores as outcomes were reported using different measurements or units. For each study, the SMD was computed as:

$$\mathrm{SMD}=\left(\text{mean}\_\text{DOMS}-\text{mean}\_\text{baseline}\right)/\mathrm{SD}\_\text{diff},$$

where SD_diff is the standard deviation of the within‐subject change. When SD_diff was reported, it was used directly. When SD_diff was not reported but a *p*‐value for the paired difference was available, the observed *t* statistic was calculated from the two‐tailed *p* and degrees of freedom (df = *n* − 1) and SMD was calculated as SMD = *t*/√*n* (Cumming 2013; Devecchi et al. 2022). When neither SD_diff nor a *p*‐value was available, SD_diff was estimated from the reported SD_pre and SD_DOMS using the standard formula:

SD_diff=√SD_pre^2+SD_DOM^2−2·r·SD_pre·SD_DOMS,

using a conservative default within‐subject correlation *r* = 0.50 (Devecchi et al. 2022). When studies presented standard errors of the mean (SEM), these were converted to SD using SD = SEM × √*n*. When only median and interquartile range (IQR) values were reported, the authors were contacted to provide the mean and SD. If no response was received after three attempts, the mean and SD were estimated using established and commonly used formulas (Wan et al. 2014).

Only studies reporting (or allowing the computation of) mean ± SD for pre‐and post‐measurements were included in the pooled analyses. Studies for which missing values could not be reliably estimated were excluded from the quantitative synthesis but were included in the narrative synthesis.

Pooled estimates were obtained using a random‐effect model with between‐study variance estimated using restricted maximum likelihood in MATLAB (Matlab 2024) (Tanriver‐Ayder et al. 2021). Heterogeneity was quantified using *I* ^2^, adopting the commonly used thresholds for interpretation (not important < 40%, moderate 30%–60%, important > 50%) (Deeks et al. 2019; Higgins et al. 2003; Hopkins et al. 2009).

For each pooled analysis, forest plots were generated illustrating study SMDs with 95% confidence intervals (CIs), study weights and a diamond indicating the pooled SMD and its CI, with marker sizes proportional to study weight. Funnel plots were generated to assess potential publication bias.

When overall heterogeneity was important (*I* ^2^ > 50%) or pooled conclusions appeared sensitive to particular studies, sensitivity analyses were conducted by removing individual studies one at a time to assess the robustness of the pooled estimate and heterogeneity statistics (Higgins et al. 2003). Moreover, if asymmetry was identified visually using funnel plots or statistically with Egger's test and Begg's rank correlation test, studies suspected of publication bias were excluded to assess whether this affected the results. Additionally, sensitivity analyses were conducted by excluding studies with poor methodological quality to assess the impact of potential biases on the results.

When a study reported results for different groups such as sexes (male/female), the study was presented twice in the meta‐analysis, once for each sex. This approach ensured that sex‐specific effects were preserved. When an outcome was reported for multiple lumbar regions or both sides (e.g., PPT at several lumbar sites), a single summary measure per study was computed by averaging region‐specific means to avoid double‐counting; this was done consistently across studies so that each study contributed one effect size per pooled analysis.

Moreover, as part of our eligibility assessment, participant characteristics, recruitment details and study authorship were cross‐checked to detect potential sample overlap across publications. When two or more studies were found to originate from the same participant cohort, only the dataset providing the most complete or methodologically robust information for the duplicated outcomes of interest was included.

SMD magnitudes were interpreted using the following descriptive thresholds: 0.0–0.2 (trivial), 0.2–0.6 (small), 0.6–1.2 (moderate), 1.2–2.0 (large), 2.0–4.0 (very large) and > 4.0 (extremely large) (Hopkins et al. 2009).

### 2.15 Confidence in Cumulative Evidence

The certainty of the evidence was assessed using the Grading of Recommendations Assessment, Development, and Evaluation (GRADE) approach (Goldet and Howick 2013; Ryan and Hill 2016). Certainty was categorized as “High”, “Moderate”, “Low”, or “Very Low” for each outcome domain defined in the previously described PICOS framework. For non‐randomized controlled trials (RCTs), the baseline rating started at “Low”. Evidence could be upgraded for a large magnitude of effect, a dose–response relationship, or when all plausible confounders would reduce the observed effect (or create a spurious effect if none was observed). Upgrading was applied by one (+1) or two (+2) levels. Possible downgrading was evaluated across five domains: risk of bias (assessed using the NIH quality assessment tool), inconsistency (differences in narrative synthesis or important heterogeneity, e.g., *I* ^2^ > 50%), indirectness (population, intervention, comparator, or outcome not directly aligned with the research question), imprecision (total sample size < 400 participants or CI crossing a clinically relevant threshold, e.g., SMD ±0.5) and publication bias (assessed through study size and funnel plot asymmetry). Serious concerns led to downgrading by one level, and very serious concerns by two levels. The authors rated the certainty of the evidence for each outcome (J.D. and E.G.‐W.). Disagreements were resolved through consultation with a third author (J.A.).

## 3 Results

### 3.1 Study Selection

**FIGURE 1:**
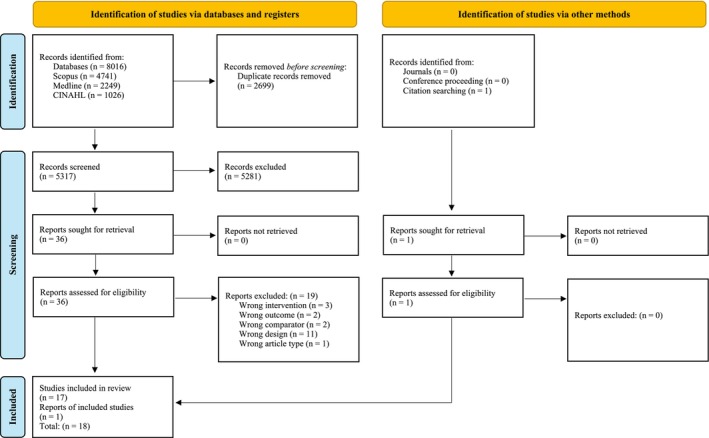
PRISMA flowchart illustrating the study selection process.

The results of the search and selection process are presented in the flowchart (Figure 1). A total of 8016 records were identified through database searches. After removing 2699 duplicates, 5317 records remained for screening. Following the screening of titles and abstracts, 36 full‐text articles were assessed for eligibility. Among these, 19 articles were excluded due to wrong intervention (e.g., DOMS not specific to the lumbar region) (*n* = 3), wrong outcome (*n* = 2), wrong comparator (*n* = 2), wrong study design (*n* = 11), or wrong article types (*n* = 1). Detailed reason for the exclusion of each study can be found in Supporting Information S2. In addition to database searching, studies were also identified through other methods. Specifically, no additional records were identified through journals (*n* = 0) or conference proceedings (*n* = 0), whereas one study was identified through reference list searching of included studies, retrieved using ProQuest and assessed for eligibility (*n* = 1). Finally, 18 studies were included in the review.

### 3.2 Characteristics of the Studies

All details regarding the included studies are provided in Supporting Information S3. Among the included studies, seven used a back‐extension protocol performed until exhaustion to induce DOMS (Bishop, Horn, George, and Robinson 2011; Bishop, Horn, Lott, et al. 2011; Horn and Bishop 2013; McPhee and Graven‐Nielsen 2019; Than et al. 2019; Trost et al. 2012, 2011), while 11 studies used a protocol requiring participants to complete predefined number of weighted back extensions (Abboud et al. 2021, 2019; Arvanitidis et al. 2024; Chen et al. 2019; Ducas et al. 2024, 2025; Houle et al. 2020; Lee et al. 2020; Mounier et al. 2025; Pano‐Rodriguez et al. 2025; Udermann et al. 2002). Moreover, one study used repeated‐bout DOMS, inducing two episodes of DOMS separated by a two‐week interval (Chen et al. 2019). For the present meta‐analyses, only the first bout was included, as the second session was potentially influenced by the residual effects of the first bout, and DOMS needed to be induced without interaction with other interventions (Chen et al. 2019). One study used both concentric and eccentric exercise protocols separated by 14 days; however, only the eccentric‐exercise data were extracted, as the concentric protocol did not induce DOMS (Lee et al. 2020). Another study reported only mean values without SDs, and we were unable to contact the authors. Therefore, these data could not be included in the meta‐analysis and are presented only in the extraction table (Supporting Information S3) (Udermann et al. 2002). Regarding outcomes, all studies assessed clinical variables (e.g., PPT, pain intensity, soreness). Seventeen studies assessed biomechanical variables: 11 examined kinetic variables related to forces and moments acting on the body, three examined kinematic variables related to movement and three examined both kinetic and kinematic variables. Additionally, six studies investigated neuromuscular variables. Regarding study design, 16 used a pre‐post design and two used a crossover design. Across all studies, a total sample of 452 (226 male, 226 female) participants were included, but two studies excluded participants who did not report any pain at the second session (*n* = 15 total) (Horn and Bishop 2013; McPhee and Graven‐Nielsen 2019). The mean age of the total sample was 23.5 ± 2.8 years.

### 3.3 Quality Assessment

The methodological quality of the included studies was rated as good for one study (Than et al. 2019), fair for 16 studies (Abboud et al. 2021, 2019; Arvanitidis et al. 2024; Bishop, Horn, George, and Robinson 2011; Bishop, Horn, Lott, et al. 2011; Chen et al. 2019; Ducas et al. 2024, 2025; Horn and Bishop 2013; Houle et al. 2020; Lee et al. 2020; McPhee and Graven‐Nielsen 2019; Mounier et al. 2025; Pano‐Rodriguez et al. 2025; Trost et al. 2011, 2012), and poor for one study (Udermann et al. 2002). Following a conservative approach, information that was not reported was considered as not done. Consequently, most studies did not meet the criterion for adequate sample size, with only six studies fulfilling this requirement (Arvanitidis et al. 2024; Chen et al. 2019; Ducas et al. 2024, 2025; Pano‐Rodriguez et al. 2025; Than et al. 2019). Additionally, all studies failed to meet the criterion related to sample representativeness, as they recruited primarily young participants, limiting the generalizability of the findings to other populations. Furthermore, none of the studies except Houle et al. (2020) met the criterion for assessor blinding, as blinding of DOMS assessment and data processing was not reported. Regarding outcomes, 12 studies were considered at risk of bias because soreness was assessed using subjective self‐report tools (e.g., 0–10 or 0–100 visual analog scales (VAS), Likert scales) that are not validated specifically for muscle soreness. In addition, two studies were considered at risk of bias because PPT was measured only once per region (Bishop, Horn, George, and Robinson 2011; Bishop, Horn, Lott, et al. 2011), or because a single trunk extension MVC value was recorded (*n* = 2) (Lee et al. 2020; Udermann et al. 2002), thereby limiting the reliability and precision of outcome assessment. A detailed assessment of risk of bias is presented in Table 2.

**TABLE 2:** Methodological quality of the included studies using the quality assessment tool for before‐after studies with no control group.

Authors (year)	1	2	3	4	5	6	7	8	9	10	11	12	Quality rating
Abboud et al. (2021)	Y	N	N	Y	NR	Y	N	NR	Y	Y	Y	NA	Fair
Abboud et al. (2019)	Y	Y	N	Y	NR	Y	N	NR	Y	Y	Y	NA	Fair
Arvanitidis et al. (2024)	Y	Y	N	Y	Y	Y	N	NR	Y	Y	Y	NA	Fair
Bishop, Horn, George, and Robinson (2011)	Y	Y	N	Y	NR	Y	Y	NR	Y	Y	N	NA	Fair
Bishop, Horn, Lott, et al. (2011)	Y	Y	N	Y	NR	Y	Y	NR	Y	Y	N	NA	Fair
Chen et al. (2019)	N	Y	N	Y	Y	Y	N	NR	Y	Y	Y	NA	Fair
Ducas et al. (2024)	Y	Y	N	Y	Y	Y	N	NR	Y	Y	Y	NA	Fair
Ducas et al. (2025)	Y	Y	N	Y	Y	Y	N	NR	Y	Y	Y	NA	Fair
Horn and Bishop (2013)	Y	Y	N	Y	NR	Y	Y	NR	Y	Y	Y	NA	Fair
Houle et al. (2020)	Y	Y	N	Y	NR	Y	N	Y	Y	Y	Y	NA	Fair
Lee et al. (2020)	Y	Y	N	Y	NR	Y	N	NR	Y	Y	N	NA	Fair
McPhee and Graven‐Nielsen (2019)	Y	Y	N	Y	NR	Y	N	NR	Y	Y	Y	NA	Fair
Mounier et al. (2025)	Y	Y	N	Y	N	Y	N	NR	Y	Y	Y	NA	Fair
Pano‐Rodriguez et al. (2025)	Y	Y	N	Y	Y	Y	N	NR	Y	Y	Y	NA	Fair
Than et al. (2019)	Y	Y	N	Y	Y	Y	Y	NR	Y	Y	Y	NA	Good
Trost et al. (2012)	Y	Y	N	Y	NR	Y	Y	NR	Y	Y	Y	NA	Fair
Trost et al. (2011)	Y	Y	N	Y	NR	Y	Y	NR	Y	CD	Y	NA	Fair
Udermann et al. (2002)	Y	N	N	Y	NR	Y	N	NR	Y	Y	N	NA	Poor

*Note:* (1) Objective clearly stated; (2) eligibility criteria described; (3) representative patient population; (4) all eligible participants enrolled; (5) sample size sufficient; (6) intervention described; (7) outcome measures specified; (8) outcome assessors blinded; (9) loss to follow up reported; (10) statistical analysis of outcomes pre‐ and post‐intervention; (11) interrupted time series design; (12) individual data used for group‐level effects.

Abbreviations: CD = cannot determine, *N* = no, NA = not applicable, NR = not reported, Y = yes.

### 3.4 Certainty of the Evidence

The analyses revealed a very low certainty of evidence regarding the effects of DOMS on all outcomes, except for soreness on Day 1, which showed a large effect size and was therefore upgraded to low (Table 3). Soreness on Day 2 also showed a large effect size, but it was not upgraded due to the high heterogeneity. As none of the included studies were RCTs, all outcomes began at a low level of certainty and were further downgraded to very low due to insufficient sample sizes (< 400 participants) (Ryan and Hill 2016), limiting confidence in the findings.

**TABLE 3:** Summary of findings and certainty of evidence (grading of recommendations assessment, development and evaluation).

Outcomes	SMD (CI)	*N* of participants (*N* of study)	Findings summarized	Certainty of the evidence (GRADE)
*Low back pain intensity*				
Day 1	1.12 [0.97; 1.27]	336 (12)	The lumbar DOMS protocol elicited pain at 1, 2, 3 and 4 days post‐exercise	Very low
Day 2	0.94 [0.77; 1.11]	218 (8)		Very low
Day 3	0.69 [0.48; 0.91]	104 (5)		Very low
Day 4	0.49 [0.31; 0.67]	136 (5)		Very low
*Soreness*				
Day 1	1.94 [1.63; 2.25]	166 (9)	The lumbar DOMS protocol elicited soreness at 1, 2, 3 and 4 days post‐exercise	Low
Day 2	1.66 [1.22; 2.11]	133 (7)		Very low
Day 3	0.88 [0.55; 1.21]	89 (5)		Very low
Day 4	0.59 [0.33; 0.85]	69 (4)		Very low
*Pressure pain threshold*				
Day 1	−0.47 [−0.64; −0.30]	156 (6)	The lumbar DOMS protocol decreased pressure pain threshold values at 1 and 2 days post‐exercise	Very low
Day 2	−0.44 [−0.63; −0.25]	116 (4)		Very low
*Trunk extension maximal voluntary contraction*				
Day 1	−0.67 [−0.90; −0.44]	207 (9)	The lumbar DOMS protocol decreased maximal voluntary contraction values at 1, 2, 3 and 4 days post‐exercise	Very low
Day 2	−0.95 [−1.32; −0.57]	133 (6)		Very low
Day 3	−0.84 [−1.46; −0.23]	41 (3)		Very low
Day 4	−0.38 [−0.75; −0.01]	93 (4)		Very low
*Full trunk flexion*				
Day 1	−0.11 [−0.34; 0.11]	77 (3)	Lumbar DOMS did not alter full trunk flexion amplitude	Very low
*Flexion relaxation ratio*				
Day 1	−0.09 [−0.31; 0.14]	77 (3)	Lumbar DOMS did not alter flexion relaxation ratio during full trunk flexion	Very low

### 3.5 Results of Syntheses

#### 3.5.1 Clinical Results

The overall quality of studies assessing clinical variables ranged from good to fair. Forest plots and meta‐analyses were conducted for outcomes related to pain, soreness and PPT, whereas a narrative synthesis was provided for the other variables, either because only a single study assessed them or due to important methodological heterogeneity. Data were grouped by time points as follows:

- Day 1 = 24–47 h post‐exercise
- Day 2 = 48–71 h post‐exercise
- Day 3 = 72–95 h post‐exercise
- Day 4 = 96–111 h post‐exercise
- Day 5 = 112–135 h post‐exercise

This categorization was used since most studies evaluated DOMS at slightly different time intervals but generally aligned with these categories. When multiple measurements were taken within the same time frame (e.g., pain assessed every 5 h), they were averaged to obtain a single value per day. Two studies from our research group (Ducas et al. 2025; Pano‐Rodriguez et al. 2025) included overlapping participants, resulting in duplicated pain, soreness and PPT data. To prevent double counting and maintain the independence of observations in the meta‐analysis, only the study with the largest sample size was included (Pano‐Rodriguez et al. 2025). Results extracted from individual studies are presented in Supporting Informations S3 and S4.

##### 3.5.1.1 Low Back Pain Intensity

**FIGURE 2:**
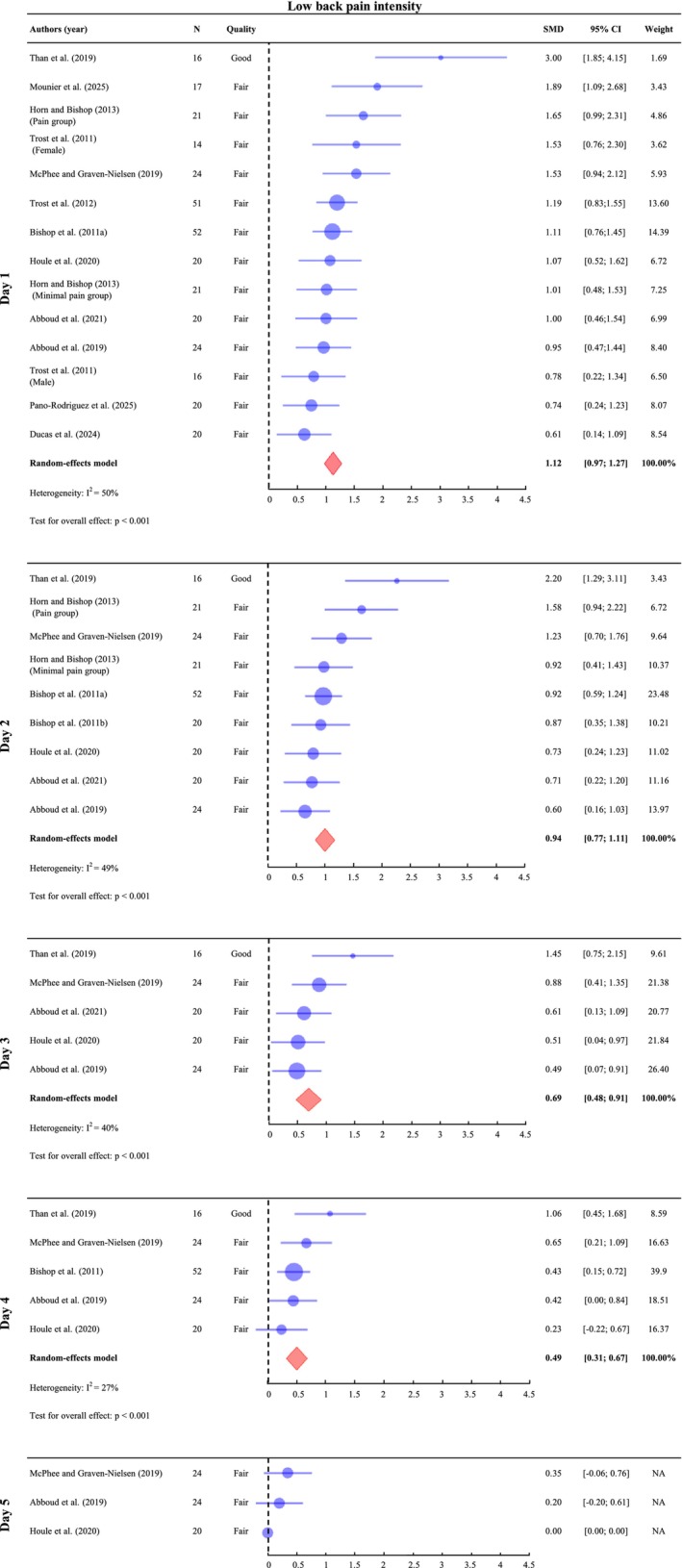
Meta‐analysis of LBP induced by the DOMS protocol from Day 1 to Day 4 post‐exercise. On Day 5, only the standardized mean differences (SMDs) of individual studies are presented because one study reported mean and standard deviation values of zero for pain, which prevented the computation of a pooled effect size. SMDs and 95% confidence intervals (95% CIs) are shown. Pain outcomes were pooled on Day 1 (24–47 h post‐exercise), Day 2 (48–71 h), Day 3 (72–95 h), Day 4 (96–111 h) and Day 5 (112–135 h). Horn and Bishop (2013) and Trost et al. (2011) reported results for different groups, which were analyzed separately.

A meta‐analysis was conducted on Day 1 (n of participants = 336), Day 2 (*n* = 218), Day 3 (*n* = 104) and Day 4 (*n* = 136). On Day 5 (*n* = 68), only the SMDs of individual studies are presented because one study reported mean and SD values of zero for pain, which prevented the estimation of variance and, consequently, the computation of a pooled effect size. Since baseline pain was an exclusion criterion in most included studies, baseline values were assumed to be zero in those cases. When baseline pain was measured, the reported values were used instead. The results indicate a significant increase in pain intensity across all assessed time points, with SMD of 1.12 (95% CI: [0.97; 1.27], *I* ^2^ = 50%, *p* < 0.001) on Day 1, 0.94 (95% CI: [0.77; 1.11], *I* ^2^ = 49%, *p* < 0.001) on Day 2, 0.69 (95% CI: [0.48; 0.91], *I* ^2^ = 40%, *p* < 0.001) on Day 3 and 0.49 (95% CI: [0.31; 0.67], *I* ^2^ = 27%, *p* < 0.001) on Day 4 (Figure 2). The effect sizes are moderate on Days 1, 2 and 3, and small on Day 4. On Day 5, Houle et al. (2020) reported a complete absence of pain in all participants. The two other studies conducted on the same day observed only low levels of pain, with SMD and CI crossing zero, indicating no significant differences between conditions (Abboud et al. 2019; McPhee and Graven‐Nielsen 2019).

##### 3.5.1.2 Soreness

**FIGURE 3:**
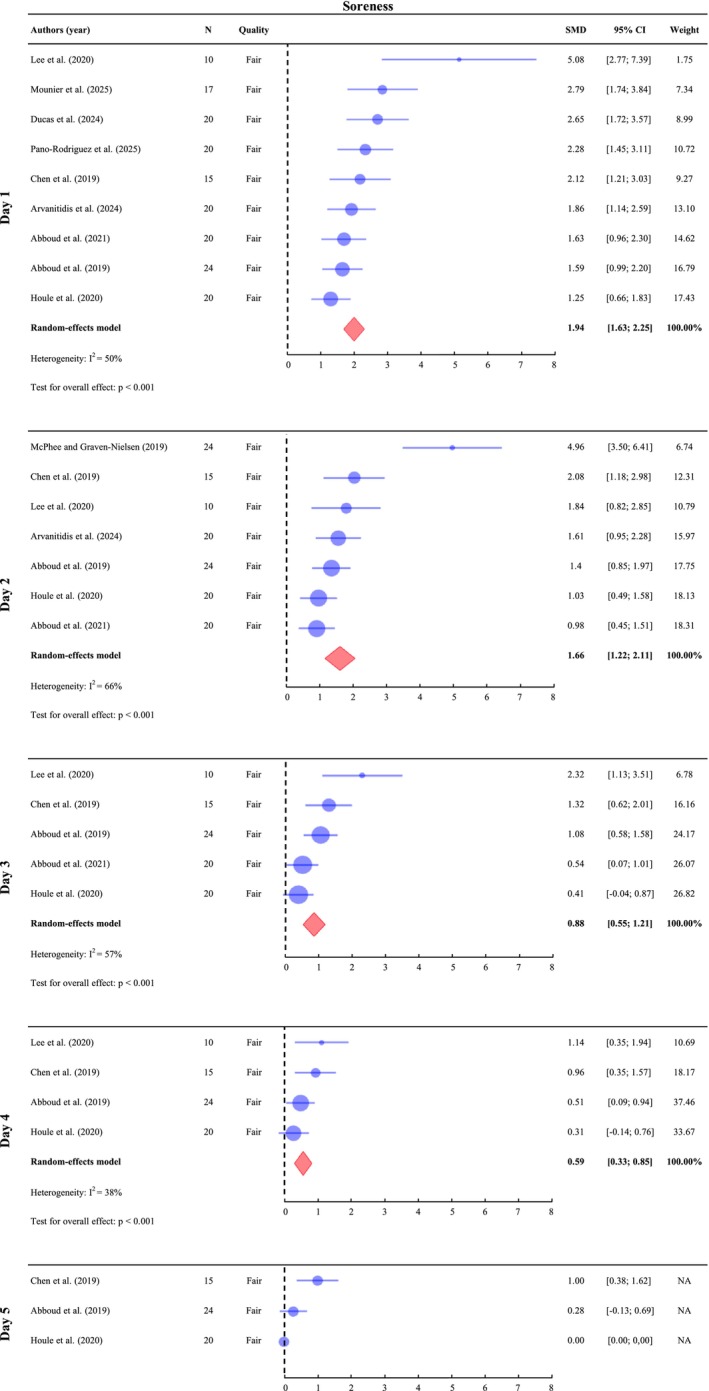
Meta‐analysis of soreness induced by the DOMS protocol from Day 1 to Day 4 post‐exercise. On Day 5, only the standardized mean differences (SMDs) of individual studies are presented because one study reported mean and standard deviation values of zero for soreness, which prevented the computation of a pooled effect size. SMDs and 95% confidence intervals (95% CIs) are shown. Soreness outcomes were pooled on Day 1 (24–47 h post‐exercise), Day 2 (48–71 h), Day 3 (72–95 h), Day 4 (96–111 h) and Day 5 (112–135 h).

A meta‐analysis was conducted on Day 1 (*n* = 166), Day 2 (*n* = 133), Day 3 (*n* = 89) and Day 4 (*n* = 69). On Day 5 (*n* = 59), only the SMDs of individual studies are presented, for the same reason described in the LBP results section. Since baseline soreness was an exclusion criterion in most included studies, baseline values were assumed to be zero in those cases. When baseline soreness was measured, the reported values were used instead. The results indicate a significant increase in soreness levels across all assessed time points, with SMD of 1.94 (95% CI: [1.63; 2.25], *I* ^2^ = 50%, *p* < 0.001) on Day 1, 1.66 (95% CI: [1.22; 2.11], *I* ^2^ = 66%, *p* < 0.001) on Day 2, 0.88 (95% CI: [0.55; 1.21], *I* ^2^ = 57%, *p* < 0.001) on Day 3 and 0.59 (95% CI: [0.33; 0.85], *I* ^2^ = 38%, *p* < 0.001) on Day 4 (Figure 3). The effect size is large on Days 1 and 2, moderate on Day 3 and small on Day 4. Significant heterogeneity is observed on Days 2 and 3, which may be attributed to variations in the DOMS induction protocols, potentially leading to different levels of soreness. On Day 5, Houle et al. (2020) reported a complete absence of soreness in all participants. One other study found no significant increase in soreness (Abboud et al. 2019), with CI crossing zero, while another study reported a small but significant level of soreness on Day 5 (Chen et al. 2019). These findings suggest that some soreness might still have been present in a few participants on Day 5, although it had been largely resolved for most individuals.

##### 3.5.1.3 Pain Sensitivity

**FIGURE 4:**
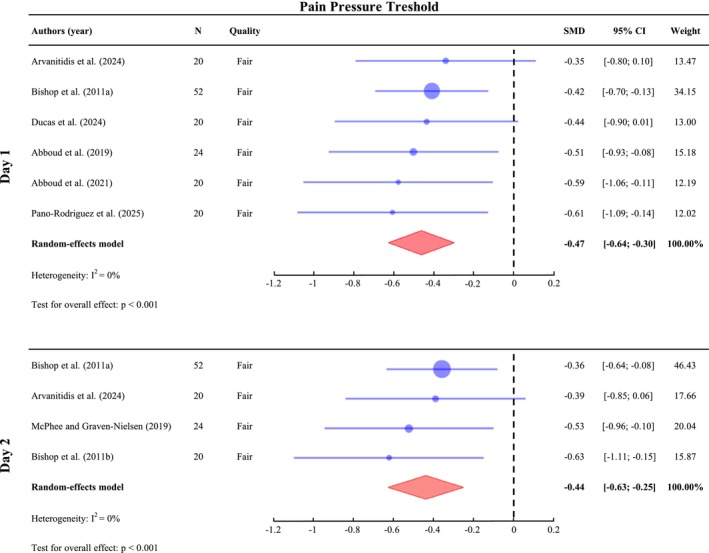
Meta‐analysis of the change in pressure pain threshold value (PPT) with DOMS. Standardized mean differences (SMDs) and 95% confidence intervals (95% CIs) are presented. PPT outcomes were pooled on Day 1 (24–47 h post‐exercise) and Day 2 (48–71 h).

A meta‐analysis was conducted on Day 1 (*n* = 156), Day 2 (*n* = 116) on PPT values to assess pain sensitivity. The results indicate a significant decrease in PPT value across all assessed time points, with SMD of −0.47 (95% CI: [−0.64; −0.30], *I* ^2^ = 0%, *p* < 0.001) on Day 1 and −0.44 (95% CI: [−0.63; −0.25], *I* ^2^ = 0%, *p* < 0.001) on Day 2 (Figure 4). The effect size is small on both Days 1 and 2 post‐DOMS. One study assessed PPT on Day 4 but did not observe a significant difference from the baseline (*p* > 0.05) (Bishop, Horn, George, and Robinson 2011). Furthermore, another study assessed regional sensitivity using a PPT heatmap and found no difference between either Day 1 or Day 2 compared to baseline (Arvanitidis et al. 2024).

##### 3.5.1.4 Pain Location

Three studies assessed DOMS pain distribution using body charts (Bishop, Horn, George, and Robinson 2011; Bishop, Horn, Lott, et al. 2011; McPhee and Graven‐Nielsen 2019). Pain area was found to increase at Day 2 post‐exercise (Bishop, Horn, Lott, et al. 2011). In one study, some participants reported back and leg pain, but none below the knee (Bishop, Horn, George, and Robinson 2011). Another study illustrated pain distribution visually without providing quantitative data (McPhee and Graven‐Nielsen 2019).

##### 3.5.1.5 Pain Unpleasantness and Quality

Two studies assessed pain unpleasantness and quality (Bishop, Horn, George, and Robinson 2011; McPhee and Graven‐Nielsen 2019). DOMS induced mild unpleasantness (2.8/10) described as “annoying” or “sore” at Day 2 (McPhee and Graven‐Nielsen 2019), and increased the affective ratio (unpleasantness/intensity) from Day 1 to Day 2 (Bishop, Horn, George, and Robinson 2011).

##### 3.5.1.6 Pain Interference and Disability

Disability and pain interference were assessed using various tools across studies (see Supporting Information Table 3 for more details). DOMS induced moderate pain interference at Day 1 (Trost et al. 2012, 2011) and mild disability at Days 1 and 2 (Bishop, Horn, George, and Robinson 2011; McPhee and Graven‐Nielsen 2019), with scores peaking at Day 1 and returning toward baseline over time (Bishop, Horn, George, and Robinson 2011).

#### 3.5.2 Biomechanical Results

The overall quality of studies assessing biomechanical variables ranged from good to fair. Forest plots and meta‐analyses were conducted for outcomes related to trunk extension MVC and full trunk flexion, whereas a narrative synthesis was provided for the other variables. Data were grouped by the same time points as for clinical variables (Days 1, 2, 3, 4 and 5). Results extracted from individual studies are presented in Supporting Informations S3 and S4.

##### 3.5.2.1 Trunk Extension Maximal Voluntary Contraction

**FIGURE 5:**
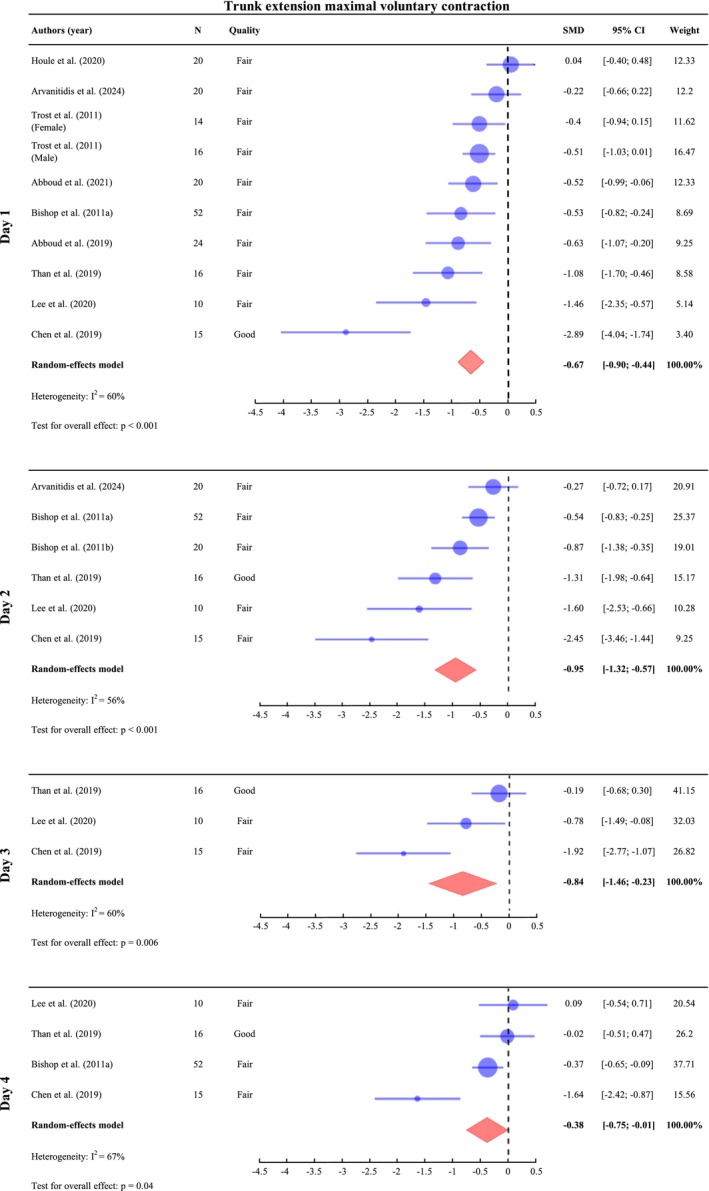
Meta‐analysis of the change in trunk extension maximal voluntary contraction (MVC) value with DOMS. Standardized mean differences (SMDs) and 95% confidence intervals (95% CIs) are presented. MVC outcomes were pooled on Day 1 (24–47 h post‐exercise), Day 2 (48–71 h), Day 3 (72–95 h) and Day 4 (96–111 h). Trost et al. (2011) reported results for different groups, which were therefore analyzed separately.

A meta‐analysis was conducted on Day 1 (*n* = 207), Day 2 (*n* = 133), Day 3 (*n* = 41) and Day 4 (*n* = 93). On Day 5, only one study assessed MVC (Chen et al. 2019), and the corresponding results are provided in Supporting Information S3. The meta‐analysis focused on concentric MVC, as only one study also assessed eccentric MVC using an isokinetic device (Arvanitidis et al. 2024). The meta‐analysis results on concentric MVC indicate a significant decrease in MVC value across all assessed time points, with SMD of −0.67 (95% CI: [−0.90; −0.44], *I* ^2^ = 60%, *p* < 0.001) on Day 1, −0.95 (95% CI: [−1.32; −0.57], *I* ^2^ = 56%, *p* < 0.001) on Day 2, −0.84 (95% CI: [−1.46; −0.23], *I* ^2^ = 60%, *p* = 0.006) on Day 3 and −0.38 (95% CI: [−0.75; −0.01], *I* ^2^ = 67%, *p* = 0.04) on Day 4 (Figure 5). The effect size is moderate on Days 1, 2 and 3 and small on Day 4. Significant heterogeneity was observed across all post‐DOMS induction protocol days, likely reflecting differences in reported soreness and pain between studies, which may influence MVC reduction. For example, on Day 1, the three studies with the largest MVC reductions reported either some of the highest average soreness scores (VAS/100 mm: 52.8 ± 10.4, SMD = 5.08, Lee et al. (2020); 61.9 ± 29.2, SMD = 2.12, Chen et al. (2019)) or the highest pain level (VAS/10: 4.8 ± 1.6, SMD = 3.00, Than et al. (2019)) (Figures 2, 3; Supporting Information S3). Meta‐regression was not possible, as some studies assessed pain while others measured soreness. Regarding eccentric MVC, no significant differences were observed between baseline and Days 1 and 2 post‐exercise (Arvanitidis et al. 2024).

##### 3.5.2.2 Torque Steadiness

One study reported increased torque steadiness during both eccentric and concentric contractions post‐exercise at Days 1 and 2 (Arvanitidis et al. 2024). In contrast, another study reported no change in concentric trunk extension torque steadiness on Day 1 post‐exercise (Pano‐Rodriguez et al. 2025).

##### 3.5.2.3 Trunk Proprioception

One study found that DOMS, during a trunk force reproduction task, did not affect trunk force reproduction accuracy (constant error, absolute error and variable errors) but significantly reduced time to peak force at Day 1 post‐exercise (Houle et al. 2020).

##### 3.5.2.4 Lumbar Stiffness

One study found that DOMS increased lumbar stiffness and reduced hysteresis at L3 at Day 1 post‐exercise (Mounier et al. 2025).

##### 3.5.2.5 Muscle Endurance

One study measured muscle endurance in seconds using the Sorensen test and found no significant changes at any time point post‐exercise (Days 1, 2, 3 and 4) (Lee et al. 2020).

##### 3.5.2.6 Postural Stability

DOMS at Day 1 did not significantly affect balance measures across bipedal (eyes open and closed) and one‐leg standing tasks (Ducas et al. 2024).

##### 3.5.2.7 Full Trunk Flexion Range of Motion

**FIGURE 6:**
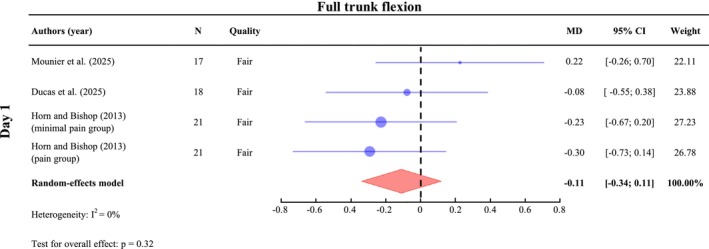
Meta‐analysis of changes in full trunk flexion following DOMS. Standardized mean differences (SMDs) and 95% confidence intervals (95% CIs) are presented. Outcomes were pooled on Day 1 (24–47 h post‐exercise). Horn and Bishop (2013) reported results for different groups, which were therefore analyzed separately.

A meta‐analysis was conducted on Day 1 (*n* = 77) on full trunk flexion range of motion (ROM) during flexion relaxation tasks. The results show no significant difference in trunk flexion ROM value Day 1 post‐exercise, with SMD of −0.11 (95% CI: [−0.34; 0.11], *I* ^2^ = 0%, *p* = 0.32) (Figure 6). One study also assessed trunk flexion ROM on Day 2 and reported a reduction in ROM in both the minimal pain and pain groups, with a greater decrease observed in the pain group (Horn and Bishop 2013). Another study also assessed lumbar and hip kinematics separately during full trunk flexion and found no significant differences between pre‐exercise and Day 1 (Ducas et al. 2025).

##### 3.5.2.8 Trunk and Hip Kinematics in Other Tasks

DOMS reduced lumbar flexion during low‐target reaching on Day 1 (Trost et al. 2012). In contrast, sagittal‐plane trunk ROM increased during submaximal eccentric contraction on Days 1 and 2 but decreased during concentric contractions on Day 2. No effects were observed for hip, thoracic and frontal/transverse‐plane movements (Arvanitidis et al. 2024), nor during high‐target reaching or trunk perturbation tasks on Day 1 (Abboud et al. 2021; Trost et al. 2012).

#### 3.5.3 Neuromuscular Results

The overall quality of studies assessing neuromuscular variables ranged from good to fair. Forest plots and meta‐analyses were conducted for the flexion relaxation phenomenon, whereas a narrative synthesis was provided for the other variables. Results extracted from individual studies are presented in Supporting Informations S3 and S4.

##### 3.5.3.1 Flexion Relaxation Phenomenon

**FIGURE 7:**
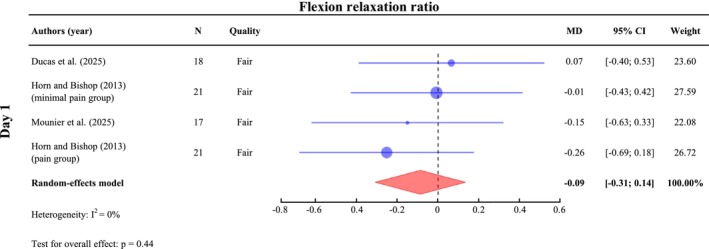
Meta‐analysis of changes in flexion relaxation ratio following DOMS. Standardized mean differences (SMDs) and 95% confidence intervals (95% CIs) are presented. Outcomes were pooled for Day 1 (24–47 h post‐exercise). Horn and Bishop (2013) reported results for different groups, which were therefore analyzed separately.

A meta‐analysis was conducted on Day 1 (*n* = 77) for flexion relaxation ratios during flexion relaxation tasks. The results show no significant difference in trunk flexion relaxation ratio value on Day 1 post‐exercise, with SMD of −0.09 (95% CI: [−0.31; 0.14], *I* ^2^ = 0%, *p* = 0.44) (Figure 7). One study assessed trunk flexion relaxation ratio on Day 2 post‐exercise and reported a decrease in the ratio only in the pain group, although this change was not statistically significant (Horn and Bishop 2013).

One study assessed the flexion relaxation phenomenon using the extension relaxation ratio and found no significant effect of DOMS at Day 1 (Mounier et al. 2025). The same study also measured trunk flexion angles, showing an increased trunk flexion angle at the onset of myoelectric silence, while no significant change was observed at the cessation angle of silence during trunk extension (Mounier et al. 2025). Another study, using high‐density surface electromyography (HDsEMG), examined the localization of the phenomenon within the lumbar region and found a significant cranial shift in the flexion relaxation response with DOMS at Day 1 (Ducas et al. 2025).

##### 3.5.3.2 Muscle Activity Amplitude and Localization

Two studies using HDsEMG found that DOMS did not significantly alter EMG amplitude or muscle activity localization during submaximal trunk contractions on Day 1 or 2 (Arvanitidis et al. 2024) or perturbation tasks on Day 1 (Abboud et al. 2021). Under DOMS, the typical trial‐by‐trial decrease in EMG reflex amplitude observed in control conditions was absent, indicating a lack of neuromuscular adaptations (Abboud et al. 2021). The same study also assessed baseline muscle activity prior to each trunk perturbation and found no effect of DOMS (Abboud et al. 2021).

##### 3.5.3.3 Median Frequency

One study assessed median frequency and its spatial distribution in the lumbar region on both sides of the trunk using HDsEMG. Median frequency values showed no significant change on Day 1 post‐exercise on either side compared to pre‐exercise (Pano‐Rodriguez et al. 2025). However, the median frequency centroids, which indicate the location of the highest median‐frequency value within the lumbar muscle, shifted after Day 1 post‐exercise, with a medial shift on the right side and a cranial shift on the left side (Pano‐Rodriguez et al. 2025).

##### 3.5.3.4 Neuromuscular Control Efficiency

The EMG‐torque relationship reflects how effectively neural drive is translated into torque. In DOMS, δ‐band coherence and erector spinae contribution decreased on Day 2 during eccentric contractions (Arvanitidis et al. 2024), indicating impaired synchronization and reduced neural drive efficiency to the erector spinae.

##### 3.5.3.5 Reflex Latency

Reflex latency was measured in one study using HDsEMG during a trunk unexpected perturbation task, with no significant effect of DOMS observed on Day 1 (Abboud et al. 2021).

### 3.6 Results Summary

The results are summarized in Table 3.

### 3.7 Sensitivity Analyses

Sensitivity analyses were conducted when asymmetry was detected either visually in funnel plots or statistically using Egger's test and Begg's rank correlation test. In such cases, studies contributing to the asymmetry were excluded to minimize the potential influence of publication bias. These exclusions did not alter the overall significance of the results. Overall, these results indicate that the main findings are robust to the exclusion of studies with potential bias or lower quality.

## 4 Discussion

### 4.1 Summary of Findings

Overall, the presence of DOMS led to significant changes in several outcome measures, including increased pain and soreness intensity, increased pain sensitivity and reduced trunk extension MVC. However, there was no significant difference observed for full trunk flexion ROM or the flexion relaxation phenomenon with or without DOMS. For other outcomes, due to the limited number of available studies, findings should be interpreted with caution and further research is needed to confirm them.

Lumbar DOMS reproduces several key features of individuals with clinical LBP, including localized pain, reduced PPT and decreased trunk muscle strength. Specifically, lumbar DOMS protocols caused a moderate increase in LBP on Days 1, 2 and 3 with a smaller effect by Day 4. Although no meta‐analysis could be conducted on Day 5, the findings suggest that pain may be resolved by that day. Overall, these pain levels are considerably lower than those typically reported in individuals with clinical LBP. For instance, individuals with acute LBP (*n* = 230) reported on average 5.66/10 pain (numerical rating scale [NRS]) (Osagie et al. 2025), while another study reported a mean score of 6.4/10 (NRS) in 304 individuals with subacute LBP (Van Der Roer et al. 2006). In chronic pain, Suzuki et al. (2020) reported a mean pain intensity of 5.54/10 (NRS) in 161 individuals with chronic LBP, while Van Der Roer et al. (2006) found a baseline pain score of approximately 6.2/10 (NRS) in 138 individuals with chronic primary LBP prior to RCT intervention. Among the studies included in the present review, the highest pain intensity was 4.8 in 16 participants (Than et al. 2019), an outlier compared with the generally lower values reported in other studies. However, one advantage of the DOMS protocol in inducing experimental pain is that, despite low pain intensity, its temporal and qualitative pain profile, especially movement‐evoked pain caused by inflammation and muscle damage, closely resembles that observed in individuals with clinical LBP (Cheung et al. 2003; Knox et al. 2022). This makes it a suitable model for evaluating pain adaptation, as it could reproduce the movement avoidance behaviour observed in individuals with clinical LBP (Meulders 2019; Osagie et al. 2025; Trost et al. 2012, 2011; Vlaeyen and Crombez 1999).

Another shared feature with individuals with clinical LBP is the increase in pain sensitivity. PPT decreased slightly on the first and second days after lumbar DOMS protocols, suggesting increased pain sensitivity. A study on individuals with acute LBP reported decreased PPT compared to controls (Klyne et al. 2018), and a meta‐analysis on individuals with chronic spinal pain (including LBP) also found decreased PPT values, reflecting increased sensitization of the painful region (Amiri et al. 2021). This increased pain sensitivity is thought to result from inflammatory processes and tissue damage in the lumbar region (Friden et al. 1983; Latremoliere and Woolf 2009). Together with the nerve growth factor experimental pain model, which can also induce pain sensitivity (Barker et al. 2020), DOMS can replicate the increased sensitivity observed in individuals with clinical LBP, a feature that most other experimental pain models fail to reproduce. This makes it possible to further explore motor adaptations resulting from increased sensitivity in a controlled environment.

Another similarity with individuals with clinical LBP is the reduction in functional performance. A transient reduction in trunk extension MVC was observed with DOMS, with moderate reductions on Days 1, 2 and 3 with a gradual return toward baseline by Day 4, although the difference remained significant. This reduction aligns with evidence from reviews on clinical LBP, which report decreased trunk muscle strength and functional capacity (Matheve et al. 2023; Steele et al. 2014). However, the underlying mechanisms may differ. In DOMS, strength loss is thought to result primarily from muscle damage (Cheung et al. 2003), whereas in individuals with clinical LBP, it is more commonly associated with muscle atrophy, altered muscle composition and neuromuscular strategies (Matheve et al. 2023; Oddsson and De Luca 2003; Steele et al. 2014). Despite these differences, the transient reduction in trunk muscle performance following DOMS provides a useful model for the temporary motor impairments often observed during episodes of acute LBP (Akebi et al. 1998; Reyes‐Ferrada et al. 2021).

One difference between individuals with lumbar DOMS and individuals with chronic LBP is the induction of muscle soreness which is less common in chronic LBP (Jensen et al. 2013). Lumbar DOMS protocols induce important soreness on Days 1 and 2 post‐exercise that gradually decreases over the next 3 days. Soreness primarily reflects physiological processes such as muscle inflammation, microtrauma and subsequent muscle repair (Cheung et al. 2003; Clarkson and Hubal 2002), whereas pain represents the perceptual and emotional interpretation of these sensory signals, which can be influenced by cognitive and contextual factors (IASP 2017). Pain in individuals with chronic LBP is typically not caused by ongoing muscle damage. Instead, in chronic LBP it is hypothesized to reflects altered central pain processing and changes in muscle structure or activation patterns (e.g., muscle atrophy, fatty infiltration) (Hodges and Danneels 2019; Matheve et al. 2023; Meier et al. 2019). As a result, the pain tends to be deep, sharp, achy or diffuse, and less associated with the tenderness and mechanical sensitivity that characterize soreness (Jensen et al. 2013). In individuals with acute LBP, by contrast, pain is often hypothesized to come from transient tissue strain associated with mechanical loading, inflammation, or protective neuromuscular adaptations (Hodges and Tucker 2011; Morris et al. 2020; O'sullivan and Lin 2014), closely resembling the mechanisms underlying DOMS. This similarity supports the use of lumbar DOMS as a more suitable experimental model for studying acute clinical LBP features rather than chronic pain LBP.

Another difference with individuals with clinical LBP is the lack of change in flexion ROM and in the flexion relaxation phenomenon with DOMS. Meta‐analyses have reported moderate reduction in trunk flexion ROM (Laird et al. 2014) and significant decrease in flexion relaxation ratio (Gouteron et al. 2022) in individuals with clinical LBP compared to pain‐free controls. These differences may be explained by the influence of psychological factors commonly observed in individuals with clinical LBP, such as task‐specific fear, which can contribute to altered motion (Christe et al. 2021; Imai et al. 2022), or by protective mechanisms, such as sustained muscle guarding and a reduced ability to relax the lumbar extensors at full flexion (Alschuler et al. 2009; Hodges and Danneels 2019). Nevertheless, it should be noted that some experimental pain models using different induction methods (e.g., heat thermode) have reported transient reductions in the flexion relaxation phenomenon (Dubois et al. 2011), suggesting that the absence of change observed with DOMS may depend on the specific characteristics of the pain model. Moreover, although speculative, the transient nature of DOMS, combined with participants' awareness of its benign and self‐limiting character, likely prevents the development of maladaptive beliefs or avoidance behaviours, which may partly explain why motor behaviours such as trunk ROM and the flexion relaxation phenomenon remained comparable to pain‐free conditions.

### 4.2 Limitations of Included Studies

Several limitations should be considered when interpreting the results of this review and meta‐analysis. Two studies excluded participants who did not report any pain during the second session (*n* = 15 total) (Horn and Bishop 2013; McPhee and Graven‐Nielsen 2019), which may have introduced selection bias and limited the generalizability of our findings. Moreover, most studies recruited young, healthy university participants, limiting the applicability of the results to older adults. In addition, not all studies assessed both pain and soreness, although these reflect distinct physiological and psychological processes and should be measured separately. The lack of validated instruments for assessing soreness, highlighted by the quality assessment, further introduces potential measurement bias. Future research should prioritize developing and validating standardized measures of soreness to improve comparability across experimental protocols.

### 4.3 Limitations of the Review

First, the review is limited by the inclusion of studies published or translated only in English or French, which may have resulted in the exclusion of relevant findings reported in other languages. Regarding meta‐analyses, methodological heterogeneity across studies, such as the exercise protocol used to induce DOMS, likely contributed to the variability in the pooled estimates. Furthermore, the limited number of studies for certain outcomes reduced statistical power and restricted the ability to perform subgroup analyses.

## 5 Conclusion

Overall, lumbar DOMS produces transient increases in pain, soreness and pain sensitivity, along with reduced trunk muscle function, closely resembling key features of acute LBP. Unlike chronic LBP, pain in both lumbar DOMS and acute LBP partly arises from transient tissue strain and inflammation rather than long‐term structural alterations or peripheral and central changes typically associated with persistent pain. Moreover, the absence of alterations in trunk flexion ROM and the flexion relaxation phenomenon indicates that DOMS does not reproduce the maladaptive movement typical of chronic LBP. Overall, these findings suggest that lumbar DOMS appears to be a suitable experimental model for studying motor adaptations to acute, movement‐evoked LBP in a controlled environment.

## Author Contributions

**Julien Ducas:** data curation, visualization, conceptualization, writing original draft, investigation, Writing – review and editing, formal analysis, methodology. **Clémentine Véret:** investigation, writing – review and editing, data curation. **Émilie Gauthier‐Wong:** investigation, writing – review and editing, data curation. **Martin Descarreaux:** conceptualization, validation, writing – review and editing. **Jacques Abboud:** methodology, funding acquisition, conceptualization, supervision, writing – review and editing.

## Funding

This research received funding from the Natural Sciences and Engineering Research Council of Canada (NSERC) (Jacques Abboud: RGPIN‐2020‐06076).

## Conflicts of Interest

The authors declare no conflicts of interest.

## Supporting information


**Data S1:** ejp70264‐sup‐0001‐SupinfoS1.docx.

**Data S2:** ejp70264‐sup‐0002‐SupinfoS2.docx.

**Data S3:** ejp70264‐sup‐0003‐SupinfoS3.xlsx.

**Data S4:** ejp70264‐sup‐0004‐SupinfoS4.xlsx.
