# Supplementary material for: The Effects of Lumbar Delayed Onset Muscle Soreness on Clinical, Biomechanical and Neuromuscular Outcomes: A Systematic Review and Meta‐Analysis
Source: Eur J Pain. 2026 Apr 11;30(4):e70264. doi: 10.1002/ejp.70264 (PMC13069876; doi:10.1002/ejp.70264)
Supplement: Supplementary file 1 — Data S1: ejp70264‐sup‐0001‐SupinfoS1.docx. [file EJP-30-0-s003.docx]

***Suplementary Material***

**Bibliographic search strategies**

Database: CINAHL

Search date: 09/12/2025

Limits applied : None

| **Concepts** | **#** | **Search terms** | **Results** |
| --- | --- | --- | --- |
| Concept 1  (non-MESH terms) | 1 | XB (“musc* soreness” OR “musc* damage” OR Myalgia OR DOMS OR “Musc* pain” OR “Musc* strain”) | 12,950 |
| Concept 1  (MESH terms) | 2 | (MH Musculoskeletal pain) | 1,245 |
| Concept 1  (Combined) | 3 | 1 OR 2 | 13,506 |
| Concept 2A  (non-MESH terms) | 4 | XB (Pain N5 (adaptation OR sensitivity OR management OR modulation OR perception OR measurement OR response OR intensity OR tolerance OR reduce OR increase OR experience OR self-reported OR Threshold OR Subjective OR Experience) ) OR ( XB (“Protective Motor Response” OR “Motor Control Adaptation” OR “Gait Adaptation” OR “Numerical Rating Scale” OR “Visual Analog Scale” OR “Functional status” OR “Pain measurement” OR EMG) ) | 114,705 |
| Concept 2B  (non-MESH terms) | 5 | XB (EMG OR ElectromyographyOR Posture OR Kinematic OR Adaptation OR “Biomechanical Phenomena” OR “Biomechanical Adaptation” OR “Biomechanical Change” OR Mobility OR “Range of motion” OR Movement OR Motion OR “Motor Adaptation” OR “Muscle strength” OR “Muscle flexibility” OR “Muscle Activation Patterns” OR “Muscle Contraction” OR “Muscle Activation Strategies” OR “Muscle Activity Redistribution” OR “Neuromuscular Adaptation” OR Kinetic* OR Force OR “Torque Adaptation”) | 293,713 |
| Concept 2  (MESH terms) | 6 | (MH Pain Threshold) OR (MH Pain Management) OR (MH Visual Analog Scaling) OR (MH McGill Pain Questionnaire) OR (MH Functional status) OR (MH Pain measurement) OR (MH Mobility) OR (MH Range of motion) OR (MH Movement) OR (MH Motion) OR (MH Muscle strength) OR (MH Electromyography) OR (MH Posture) OR (MH Kinematic) OR (MH Muscle Contraction) OR (MH Kinetics) | 237,310 |
| Concept 2  (Combined) | 7 | 4 OR 5 OR 6 | 514,052 |
| Concept 3  (non-MESH terms) | 8 | XB (Back OR Lumbar OR “Paraspinal muscle” OR Trunk OR “Lumbosacral region” OR Spine) | 160,231 |
| Concept 3  (MESH terms) | 9 | (MH Back) OR (MH Spine) | 16,467 |
| Concept 3  (Combined) | 10 | 8 OR 9 | 165,494 |
| Combination of concepts | 11 | 3 AND 7 AND 10 | 1,026 |

Database: Scopus

Search date: 09/12/2025

Limits applied : None

| **Concepts** | **#** | **Search terms** | **Results** |
| --- | --- | --- | --- |
| Concept 1  (non-MESH terms) | 1 | TITLE-ABS-KEY ( "musc* soreness" OR "musc* damage" OR myalgia OR doms OR "Musc* pain" OR "Musc* strain" ) | 116,194 |
| Concept 1  (MESH terms) | 2 | NA |  |
| Concept 1  (Combined) | 3 | 1 OR 2 |  |
| Concept 2A  (non-MESH terms) | 4 | TITLE-ABS-KEY ( “Pain Threshold” OR “Pain sensitivity” OR “Pain Adaptation” OR “Pain N5 Adaptation” OR “Pain Management” OR “Pain Modulation Strategies” OR “Pain Perception” OR “Pain Response” OR “Pain Intensity” OR “Pain Tolerance” OR “Reduce Pain” OR “Increase Pain” OR “Subjective Pain Experience” OR “Self-Reported Pain” OR “Protective Motor Response” OR “Motor Control Adaptation” OR “Gait Adaptation” OR “Numerical Rating Scale” OR “Visual Analog Scale” OR “Functional status” OR “Pain measurement” ) | 416,980 |
| Concept 2B  (non-MESH terms) | 5 | TITLE-ABS-KEY ( emg OR electromyography OR posture OR kinematic OR adaptation OR “Biomechanical Phenomena” OR “Biomechanical Adaptation” OR “Biomechanical Change” OR mobility OR “Range of motion” OR movement OR motion OR “Motor Adaptation” OR “Musc* strength” OR “Musc* flexibility” OR “Musc* Activation Patterns” OR “Musc* Contraction” OR “Musc* Activation Strategies” OR “Musc* Activity Redistribution” OR “Neuromuscular Adaptation” OR kinetic OR force OR “Torque Adaptation” ) | 7,798,295 |
| Concept 2  (MESH terms) | 6 | NA |  |
| Concept 2  (Combined) | 7 | 4 OR 5 OR 6 | 8,147,267 |
| Concept 3  (non-MESH terms) | 8 | TITLE-ABS-KEY ( trunk OR lumbar OR back OR “Back musc*” OR “Paraspinal musc*” OR “Lumbosacral region” OR spine ) | 1,593,488 |
| Concept 3  (MESH terms) | 9 | NA |  |
| Concept 3  (Combined) | 10 | 8 OR 9 |  |
| Combination of concepts | 11 | 1 AND 7 AND 10 | 4,741 |

Database: MEDLINE

Search date: 09/12/2025

Limits applied : None

| **Concepts** | **#** | **Search terms** | **Results** |
| --- | --- | --- | --- |
| Concept 1  (non-MESH terms) | 1 | XB (“musc* soreness” OR “musc* damage” OR DOMS  OR “musc* pain” OR “musc* strain” OR Myalgia OR “Musculoskeletal Pain”) | 45,419 |
| Concept 1  (MESH terms) | 2 | (MH Myalgia) OR (MH Musculoskeletal Pain) | 8,477 |
| Concept 1  (Combined) | 3 | 1 OR 2 | 48,283 |
| Concept 2A  (non-MESH terms) | 4 | XB (Pain N5 (adaptation OR sensitivity OR management OR modulation OR perception OR measurement OR response OR intensity OR tolerance OR reduce OR increase OR experience OR self-reported OR Threshold OR Subjective OR Experience) ) OR ( XB (“Protective Motor Response” OR “Motor Control Adaptation” OR “Gait Adaptation” OR “Numerical Rating Scale” OR “Visual Analog Scale” OR “Functional status” OR “Pain measurement” OR EMG) ) | 295,402 |
| Concept 2B  (non-MESH terms) | 5 | XB (EMG OR Electromyography OR Posture OR Kinematic OR Adaptation OR “Biomechanical Phenomena” OR “Biomechanical Adaptation” OR “Biomechanical Change” OR Mobility OR “Range of motion” OR Movement OR Motion OR “Motor Adaptation” OR “Musc* strength” OR “Musc* flexibility” OR “Musc* Activation Patterns” OR “Musc* Contraction” OR “Musc* Activation Strategies” OR “Musc* Activity Redistribution” OR “Neuromuscular Adaptation” OR Kinetic OR Force OR “Torque Adaptation”) | 2,080,145 |
| Concept 2  (MESH terms) | 6 | (MH Pain Threshold) OR (MH Functional status) OR (MH Pain measurement) OR (MH Electromyography) OR (MH Posture) OR (MH Biomechanical Phenomena) OR (MH Range of motion) OR (MH Movement) OR (MH Muscle strength) OR (MH Pain Management) OR (MH Pain Perception) | 518,713 |
| Concept 2  (Combined) | 7 | 4 OR 5 OR 6 | 2,544,037 |
| Concept 3  (non-MESH terms) | 8 | XB (trunk OR Lumbar OR back OR “Back musc*” OR “Paraspinal musc*” OR “Lumbosacral region” OR Spine) | 572,487 |
| Concept 3  (MESH terms) | 9 | (MH Back) OR (MH Back muscles) OR (MH Paraspinal muscles) OR (MH Lumbosacral region) OR (MH Spine) | 57,188 |
| Concept 3  (Combined) | 10 | 9 OR 10 | 596,743 |
| Combination of concepts | 11 | 3 AND 7 AND 10 | 2,249 |

**Supplementary material 1.**Bibliographic search strategies
