# Supplementary material for: The Effects of Lumbar Delayed Onset Muscle Soreness on Clinical, Biomechanical and Neuromuscular Outcomes: A Systematic Review and Meta‐Analysis
Source: Eur J Pain. 2026 Apr 11;30(4):e70264. doi: 10.1002/ejp.70264 (PMC13069876; doi:10.1002/ejp.70264)
Supplement: Supplementary file 2 — Data S2: ejp70264‐sup‐0002‐SupinfoS2.docx. [file EJP-30-0-s004.docx]

| **Bishop (2011)** | Wrong study design (longitudinal study) |
| --- | --- |
| **Bishop (2012)** | Wrong article type (plenary article) |
| **Boissoneault (2020)** | Wrong study design (observational study) |
| **Brandl (2023)** | Wrong study design (matched-pair study) |
| **Brandl (2024a)** | Wrong study design (randomised controlled trial) |
| **Brandl (2024b)** | Wrong study design (secondary analysis study, matched pair design) |
| **Bush (2021)** | Wrong study design (longitudinal study) |
| **Chen (2020)** | Wrong outcome (blood markers) |
| **Hjortskov (2005)** | Wrong study design (matched-pair study) |
| **Huang (2019)** | Wrong study design (parallel-group experimental design) |
| **Huang (2022)** | Wrong outcome (blood markers) and duplicate data for MVC and soreness, as the study included the same participants as Chen (2019). |
| **Larsen (2017)** | Wrong comparator (no measures of baseline vs DOMS) |
| **Lo Vecchino (2015)** | Wrong comparator (no measures of baseline vs DOMS in the lumbar region) |
| **Margoni (2025)** | Wrong intervention (not specific to the lumbar region) |
| **Mayer (2006)** | Wrong study design (Randomized controlled trial) |
| **Ozden (2024)** | Wrong intervention (DOMS induce in the trunk flexor muscle) |
| **Szikszay (2020)** | Wrong study design (Randomized controlled trial) |
| **Solis (2016)** | Wrong article type (thesis) |
| **Travers (2025)** | Wrong study design (Randomized controlled trial) |
| **Weakley (2024)** | Wrong intervention (not specific to the lumbar region) |

**Supplementary material 2.** Reason for exclusion
